# Anogenital Distance in Turkish Newborns

**DOI:** 10.4274/jcrpe.v3i3.24

**Published:** 2011-09-09

**Authors:** Behzat Özkan, Belkıs Konak, Atilla Çayır, Murat Konak

**Affiliations:** 1 Atatürk University Faculty of Medicine, Department of Pediatric Endocrinology, Erzurum, Turkey; 2 Atatürk University Faculty of Medicine, Department of General Pediatrics, Erzurum, Turkey; +90 532 513 22 99 ozkan.behzat@gmail.com

**Keywords:** Anogenital distance, newborn, measurement

## Abstract

**Objective:** Anogenital distances are considered to be a sensitive indicator of external genitalia exposure to factors such as anti-androgens, and/or endocrine distruptors during the prenatal period. Exposure to such factors can lead to changes in the anogenital measurements (AGM) of newborn infants. These measurements can be used to predict masculinization of the external genitalia in healthy newborns. The goal of this study was to determine normal values for AGM in Turkish newborns of both genders.

**Methods:** One hundred fifteen female and 135 male term newborns with no congenital defects were included in this study. A well-trained observer measured the anogenital distance by using a sliding Caliper graduated in millimeters. Anogenital distance was measured from the center of the anus to the posterior convergence of the fourchette in females and from the center of the anus to the junction of the smooth perineal skin with the base of the scrotum in males.

**Results:** Anogenital distance in males and females was 23±0.6 mm and 10.3±0.2 mm, respectively. There were significant differences in  anogenital distance values between male and female newborns (p<0.05).

**Conclusion:** The findings of this study provide data that can be used as reference standards with regard to AGM of the posterior genital structures in Turkish male and female newborns. These data will also serve in postnatal evaluations to determine the effects of prenatal exposures to factors affecting development of genitalia.

**Conflict of interest:**None declared.

## INTRODUCTION

Genetic, hormonal and environmental factors all play an important role in the development of the external genital phenotype. Exposure during intrauterine life to environmental factors such as androgens, anti-androgens and endocrine disruptors causes alterations in the hormone signals that can disrupt the normal a activity and the end-organ response, leading  to abnormalities of the genital phenotype.  The abnormalities may be associated with changes in the anogenital measaurements (AGM) obtained during the neonatal period. These measurements can be used to predict masculinization of the external genitalia or to evaluate the risk for abnormal development in healthy newborns (1,2,3). In this study, we aimed to determine the normal values for AGM in Turkish newborns of both genders. 

## MATERIALS AND METHODS

One hundred fifteen female and 135 male newborns, born at term between May 2009 and August 2009, were included in this study. The nature of the research was explained to the parents and written consent was obtained before all evaluations.

A questionnaire was used to determine mother’s age, last menstrual period, parental occupation and education level. Parity, gravida, mode of delivery, and whether the delivery was a single or multiple births were also noted. A detailed maternal history  including questioning about presence of liver and kidney diseases, diabetes, preeclampsia, hypertension, heart disease, psychiatric disease or epilepsy was taken. Mothers with chronic diseases, poor weight gain during pregnancy, and those who were on medication were excluded from the study.  In addition, newborns with genital anomalies such as hypospadias, undescended testis, chordee, hydrocele, imperforate anus, and those with systemic diseases were also excluded from the study.

**Techniques of Measurement**

**Figures 1:**
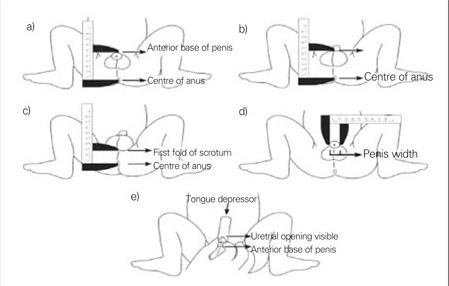
Figure 1. The distances a) from the anterior base of the penis to the center of the anus  (AGD1), (b) from the posterior base of the penis to the center of the anus  (AGD2), (c) from the posterior base of the scrotum to the center of the anus  (ASD), (d) penis width (PW), (e) stretched penis length (PL) (4)

The measurements were obtained with the newborns lying on their back (Figures 1 and 2) on a smooth surface with the legs spread and flexed from the knees, by two persons: one holding the baby in place, while the other took the measurement. To minimize subjective inter-investigator measurement variation, at the start of the study, two research assistants measured 15 male and 15 female newborns. Each took independent measurements on the same newborns on the same day but at a different time, using the same measurement technique and the same materials (Figure 1). When the measurements obtained by both researchers were compared, no statistically significant difference was noted (p>0.05). Then, three repeated measurements were performed on the same number of male and female newborns and, again no statistically significant difference was observed among the measurements (p>0.05). Therefore, the study was continued with a single researcher taking the measurements. Each newborn was placed in the same position, a single measurement was obtained and this value was recorded as the final measurement.  All measurements were made in millimeters (mm). A sliding caliper graduated in millimeters was used for the measurements (Figure 1).  

**AGM in Boys**

The following distances were measured: a) between the anterior base of the penis and the center of the anus (AGD1), b) between the posterior base of the penis and the center of the anus  (AGD2), and c) between the posterior base of the scrotum and the center of the anus  (ASD). In addition, the dimensions of the penis were measured as shown in Figures 1d and 1e. With the caliper placed at the ramus pubis, the preputium was recorded.  The diameter of the penis was measured at the widest part of the shaft. 

**AGM in Girls **

The following distances were measured and recorded: a) between the center of the anus and the beginning of the mucosa of the posterior commissure (AF), b) the beginning of the mucosa of the posterior commissure and the base of the clitoris (FC), and c) the center of the anus and the base of the clitoris (AC). The length of the clitoris was measured with a caliper and recorded.

## STATISTICAL ANALYSIS

SPSS for Windows 11.0 was used for the analysis. The results were expressed as means and standard deviations (SD), frequencies and percentiles. The student’s t-test was used for the comparisons. When more than two groups were present, a one-way ANOVA test was performed. The least significant difference (LSD) test was applied to assess group differences. For the relationship among parameters, the Pearson’s correlation test was used. For comparison of qualitative data, the chi-square test was used. The results were evaluated at the 95% confidence level and significance was set at p<0.05. 

## RESULTS

A total of 250 full-term newborns [135 males (54.0%) and 115 females (46.0%)] were included in this study. The male/female ratio was 1.17. The age and anthropometric characteristics of the infants are shown in Table 1. The gestational age of the newborns ranged from 38 to 42 weeks; the mean gestational age for females was 39.8±1.4 weeks and that for males was 39.9±1.3  weeks. There was no statistically significant difference between the two groups  (p=0.44). Table 2 lists the genital measurements for the male newborns and Table 3 the  values for the female newborns. In both genders, there were no statistically significant correlations between gestational week or mother’s body weight or height values and the genital measurements (p>0.05). However, there were significant correlations between the infants’ body measurements (body weight, length, and head circumference) and the genital measurements (p<0.05).In the female infants, a strong correlation was found between the length of the clitoris and the FC distance (r=0.93, p<0.05). When the AF, FC and AC distances were compared, there was a statistically significant negative correlation between the AF and FC distances (r=-0.50, p<0.05).  In the males, statistically significant positive correlations were found between AGD1 and AGD2, AGD1 and ASD, AGD2 and penis length. The strongest correlation observed was between AGD1 and AGD2 (r=0.86, p<0.05). There was also a strong correlation between penis length and AGD1 (r=0.92, p<0.05).

**Table 1:**
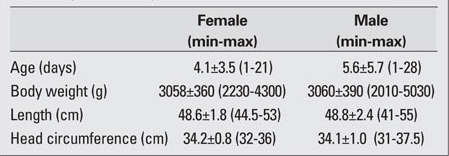
Table 1. Age and anthropometric measurements of the newborn

**Table 2:**
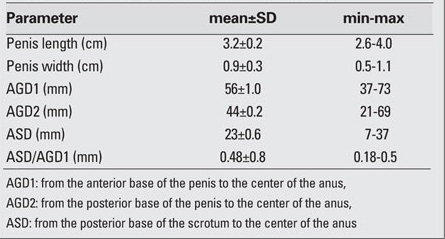
Table 2. Genital measurements in male newborns (n=135)

**Table 3:**
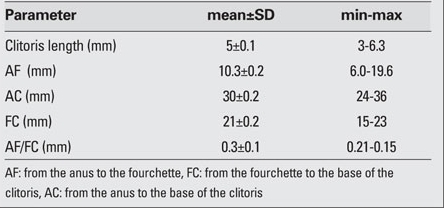
Table 3. Genital measurements in female newborns (n=115)

## DISCUSSION

Measurements of anogenital distances were reported in previous studies (4,6,7). However, the methods used for males and females differ in these prior studies.  The distances reported are: for females - between the center of the anus and the beginning of the mucosa of the posterior commissure  and for males - between the center of the anus and the base of the scrotum (4,5). 

The accuracy of the AGM can be affected by the apparatus used for the measurements, by the number of measurements and by the experience of the person taking the measurements, as well as by the number of investigators involved in taking the  easurement. Flexible tape measurements can increase the margin of error. Therefore, for improved accuracy, the adjustable vernier caliper, which is a rigid apparatus, was used in this study.Our results showed that in females, the mean AF distance was 10.3 mm. In males, the mean ASD distance was 23.0 mm. The ratio of female/male anogenital distance was 2.2. In the measurements obtained by Orish et al (6) who used flexible tapes, the AGM mean was reported as 25.8 mm in females and 30.2 mm in males. In the study by Thankamony et al (7) on 455 males and 426 females term newborns, the AGM was determined by the same distances with the vernier caliper. In that study, the AGM average was 9.1 mm in females and 19.8 mm males. Salazar et al (8), using the vernier caliper in full-term males and females, found that the AGM for the AF distance was 11 mm in females and the SA distance was 22 mm in males. In a study on  Turkish newborns, using flexible tape measurements, Oguz Kutlu et al (9) reported that the mean  AGM was 131 mm for females and 25.8 mm for males. Similar to the results of this study, anogenital distance in the girls was half that in the boys. Table 4 shows comparative data from different studies on anogenital distance measurements. Table 4 shows that the AGMs in male and female newborns can be affected by the measurement method used. It is known that the results can also be influenced by the number of measurements, experience of the person taking them, and also by the availability of an assistant during the measurement. However, the reported differences in AGM may be due to the methods used for measurement as well as to variations among different ethnic and genetic populations (7,8). 

There were no prematurely born babies in our sample and the results did not show a relationship between  gestational age week and AGMs.  AF, FC and AC in the female infants and AGD1, AGD2 and ASD in the males increased as the values of baby’s body weight, height and head circumference became greater. These findings are consistent with those  reported by Riquer et al (4) and Carlos et al (5)  

The mean length of the neonatal clitoris was 5.0±0.1 mm. The measurements obtained in prior studies were 5.02±1.68 mm reported by Oguz Kutlu et al (9) and 5.87±1.48 mm by Phillip et al (10).   The mean length of the neonatal penis was 3.2±0.2 cm. Table 5 shows a comparison of the mean  length of the penis between the present and other studies. 

Although different measurement methods were used, the results of the measurements of the clitoral and penile length obtained in this study are consistent with the  findings of previous studies. Variations in the results reported can be due to genetic and ethnic factors as well as to the  measurement methods used and the age of the newborn at the time of the measurement. Currently, the effects of environmental factors on  normal endocrine development and function are under investigation  (14,15,16). Reports have suggested that the effects of compounds such as polychlorinated biphenyl, dibutyl phthalate, diethylhexyl phthalate depend on age at exposure, as well as dose and duration of the exposure. Prior reports have shown that the younger the age of  exposure, the higher the risk of undesirable effects (2,3,12,14). Undesirable effects include shortening or  elongation of the AGM and reduction of the size of the testes, scrotum and penis (17).

In conclusion, the genital phenotype is affected by many factors. Determination of standard measurements is important, since evaluations can determine variations from the norm. The findings of this study provide data that can be used for standards with regard to the anogenital  distance of the posterior genital structures in Turkish male and female newborns.  This data can be used in postnatal evaluations that aim to determine the effects of exposures during the prenatal period. 

**Acknowlegement:** We would like to thank to Prof. Dr. Selim Kurtoglu, who is a neonatologist and pediatric endocrinologist, for his  valuable contribution to this study. 

**Table 4:**
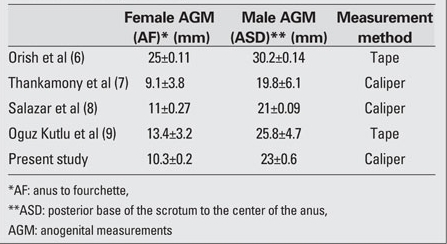
Table 4. Comparison of AGM measurements according to the measurement method used (mean±SD)

**Table 5:**
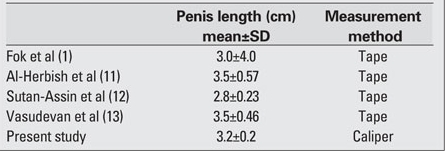
Table 5. Comparison of the mean length of the neonatal penis
